# Expanding Alternative Splicing Identification by Integrating Multiple Sources of Transcription Data in Tomato

**DOI:** 10.3389/fpls.2019.00689

**Published:** 2019-05-28

**Authors:** Sarah Clark, Feng Yu, Lianfeng Gu, Xiang Jia Min

**Affiliations:** ^1^Department of Biological Sciences, Youngstown State University, Youngstown, OH, United States; ^2^Department of Computer Science and Information Systems, Youngstown State University, Youngstown, OH, United States; ^3^Basic Forestry and Proteomics Center, College of Forestry, Fujian Agriculture and Forestry University, Fuzhou, China

**Keywords:** alternative splicing, gene expression, tomato, mRNA, plant, *Solanum lycopersicum*, transcriptome

## Abstract

Tomato (*Solanum lycopersicum*) is an important vegetable and fruit crop. Its genome was completely sequenced and there are also a large amount of available expressed sequence tags (ESTs) and short reads generated by RNA sequencing (RNA-seq) technologies. Mapping transcripts including mRNA sequences, ESTs, and RNA-seq reads to the genome allows identifying pre-mRNA alternative splicing (AS), a post-transcriptional process generating two or more RNA isoforms from one pre-mRNA transcript. We comprehensively analyzed the AS landscape in tomato by integrating genome mapping information of all available mRNA and ESTs with mapping information of RNA-seq reads which were collected from 27 published projects. A total of 369,911 AS events were identified from 34,419 genomic loci involving 161,913 transcripts. Within the basic AS events, intron retention is the prevalent type (18.9%), followed by alternative acceptor site (12.9%) and alternative donor site (7.3%), with exon skipping as the least type (6.0%). Complex AS types having two or more basic event accounted for 54.9% of total AS events. Within 35,768 annotated protein-coding gene models, 23,233 gene models were found having pre-mRNAs generating AS isoform transcripts. Thus the estimated AS rate was 65.0% in tomato. The list of identified AS genes with their corresponding transcript isoforms serves as a catalog for further detailed examination of gene functions in tomato biology. The post-transcriptional information is also expected to be useful in improving the predicted gene models in tomato. The sequence and annotation information can be accessed at plant alternative splicing database (http://proteomics.ysu.edu/altsplice).

## Introduction

Understanding the transcriptome diversity and gene expression dynamics is critical for developing methods for further improving the quantity and quality of plant products. Tomato (*Solanum lycopersicum*), one of the important fruit and vegetable crops, has its genome being completely sequenced. The complete genome of the inbred tomato cultivar “Heinz 1706” approximately has a size of 900 megabases (Mb) with a total of 34,727 protein-coding genes predicted (Tomato Genome Consortium, 2012). Recently updated release of the tomato genome assembly (SL3.0, ITAG3.2 release) contains 35,768 gene models^1^. Since the release of the tomato complete genome sequences, large amounts of RNA-seq data in tomato have been generated in a number of research projects from tissues at variable different developmental stages, growing under different conditions, or challenged with different pathogens, as well as comparative transcriptome analysis with wild tomato plants (Kumar and Khurana, 2014). Mapping RNA-seq data, generated in domesticated and wild tomato plants under various conditions or treatments, to the released complete genome sequences as a reference genome has significantly contributed to our understanding the transcriptome complexity and regulations including differential gene expressions and alternative splicing (AS) in tomato (Wang K. et al., 2016; Dai et al., 2017).

A gene can be transcribed to form two or more RNA transcripts using the process of AS in intron containing eukaryotic organisms (Reddy et al., 2013), thus, significantly increasing the diversities of mRNAs and proteins in the organism. AS commonly occurs in eukaryotes including protists, fungi, plants, and animals (McGuire et al., 2008). Four basic types of AS including exon skipping (ExonS), alternative donor site (AltD), alternative acceptor (AltA) site, and intron retention (IntronR) were commonly found (Wang and Brendel, 2006; Sablok et al., 2011). Various complex types can be formed by combination of basic events (Sablok et al., 2011). While these basic types can be found in all kingdoms of eukaryotes, ExonS is most prevalent event in animals including humans and IntronR is the dominant event in plants (McGuire et al., 2008), suggesting that the splicing mechanisms may be different in animals and plants. Numerous experimental results showed AS plays important roles in many biological processes in plants such as photosynthesis, defense responses, flowering timing, responses to stresses, etc., (Reddy et al., 2013; Staiger and Brown, 2013). AS isoforms may or may not be functional. The functional isoforms may encode distinct functional proteins and the non-functional isoforms are degraded by a process known as nonsense-mediated decay (NMD) (Lewis et al., 2003; Xu et al., 2014).

Alternative splicing has been examined in a number of plant species including the model species, *Arabidopsis thaliana*, crop plants including rice, maize, sorghum, etc., (Zhang C. et al., 2016). Due to the differences in the amounts of available gene expression data in different plant species, the estimated AS rates vary tremendously from below 10% to ∼70% in intron-containing genes (Zhang C. et al., 2016; Min, 2017). For example, due to relatively large amounts of transcription data available in Arabidopsis, it was estimated that ∼60–70% of multi-exon genes undergoing AS (Filichkin et al., 2010; Marquez et al., 2012; Yu et al., 2016; Zhang R. et al., 2017). Other well analyzed plant species were rice (*Oryza sativa*) (Wang and Brendel, 2006; Min et al., 2015; Wei et al., 2017; Chae et al., 2017; Zhang et al., 2018), maize (*Zea mays*) (Thatcher et al., 2014; Min et al., 2015; Thatcher et al., 2016; Mei et al., 2017; Min, 2017), and sorghum (*Sorghum bicolor*) (Panahi et al., 2014; Min et al., 2015; Abdel-Ghany et al., 2016); fruit plants such as grape (*Vitis vinifera*) (Vitulo et al., 2014; Sablok et al., 2017), and fiber plants such as cotton (*Gossypium raimondii*; *G. barbadense*; *G. davidsonii*, and *G. hirsutum*) (Li et al., 2014; Min, 2018; Wang et al., 2018a; Zhu G. et al., 2018). However, the above mentioned species are just few examples of AS analysis in plants, not an exhaustive list of all plants AS work. Further, genome-wide conserved AS events in flowering plant species as well as in monocot species have also been analyzed (Chamala et al., 2015; Mei et al., 2017).

Transcriptome analysis in plants have been carried out intensively using recently developed RNA-sequencing (RNA-seq) technology. A number of well-designed experiments in genome-wide transcriptome analysis for identifying differentially expressed genes and/or AS in tomato, a model plant specifically for fruit development, have been reported in recent few years. These studies include comparative transcriptome analysis of domesticated tomato for identifying differentially expressed genes in different tissues (Lopez-Casado et al., 2012; Koenig et al., 2013; Zouine et al., 2014; Sun and Xiao, 2015; Sundaresan et al., 2016; Wang K. et al., 2016; Zhang Y. et al., 2017), diurnal transcriptome changes (Higashi et al., 2016), global transcriptome profiles of tomato leaf responses to exogenous ABA or cytokinin (Wang et al., 2013; Shi et al., 2013), root transcriptome regulations in response to different plant hormone cytokinin and auxin (Gupta et al., 2013), transcriptome profiles with a focus on fruit development or in different fruit tissues (Ye et al., 2015; Zhang S. et al., 2016; Dai et al., 2017; Ezura et al., 2017) and fruit chilling tolerance (Cruz-Mendívil et al., 2015). RNA-seq data were also collected for analysis of differential gene expressions in response to tomato yellow leaf curl virus (TYLCV) infection in the TYLCV-resistant (R) breeding line and TYLCV-susceptible breeding line (Chen et al., 2013), in response to tobacco rattle virus (TRV) (Zheng et al., 2017), *Pseudomonas syringae* (Yang et al., 2015; Worley et al., 2016), *Xanthomonas perforans* (Du et al., 2015), *Cladosporium fulvum* (Xue et al., 2016), *Colletotrichum gloeosporioides* (Alkan et al., 2015), *Verticillium dahlia* (Tan et al., 2015), *Meloidogyne incognita* (root-knot nematode) (Shukla et al., 2018) and in arbuscular mycorrhiza inoculated and control plants (Zouari et al., 2014). Transcriptome analysis was also carried out with mutants including high pigment mutant (Tang et al., 2013) and SIEIN2-silenced tomato (ethylene insensitive 2) mutant which had a non-ripening phenotype (Wang R.H. et al., 2016).

The aforementioned RNA-seq projects generated large amounts of RNA-seq data in tomato, which provide an unprecedented opportunity for integrating these transcriptome data with publicly available expressed sequence tag (EST) and mRNA sequences for identifying alternatively spiced genes in tomato. However, the integrated study for AS events in tomato has not been reported. Thus, the aim of the current work is to maximize AS identification and generate a comprehensive catalog of alternatively spliced genes and AS events in tomato by integrating ESTs, mRNAs, and RNA-seq data available in public databases. The identified alternatively spliced genes and AS events with detailed annotation information in the work are expected to provide a solid resource for tomato researchers for further detailed functional analysis of these genes in tomato growth and development including fruit production.

## Materials and Methods

### Genome, EST, and mRNA Sequence Datasets

Tomato genome sequences (version SL3.0) and associated annotation files (ITAG3.20) were downloaded from the International Tomato Genome Sequencing Project (see text footnote 1). Using “*Solanum lycopersicum*” as “organism” we downloaded 300,665 EST sequences and 53,613 mRNA sequences of tomato from EST and nucleotide database at the National Center for Biotechnology Information (NCBI).

We used a procedure well implemented in our previous analysis for cleaning the data (Min et al., 2015; Min, 2018). The procedure used EMBOSS trimmest tool for trimming the polyA or polyT end (Rice et al., 2000), BLASTN search against UniVec and *E. coli* database for removal of vector and *E. coli* contaminants, and BLASTN search against the plant repeat database for removal of the repetitive sequences including transposable elements. A total of 350,141 cleaned EST and mRNA sequences were obtained and combined with 39,095 transcript sequences generated by Scarano et al. (2017) and 250,676 transcript sequences generated by Wang K. et al. (2016). Thus a total of 639,912 sequences were used for assembling using CAP3 (Huang and Madan, 1999). A total of 452,672 putative unique transcripts including 27,791 contigs and 424,881 singlets were obtained for mapping to tomato genome sequences.

### Mapping Assembled Transcripts to the Genome

We used ASFinder to map the assembled transcripts tomato genome sequences (Min, 2013). We applied the threshold values as reported previously (Walters et al., 2013). Mapped transcripts having an intron size>100 kb were removed for AS identification in order to avoid chimeric transcripts.

### RNA-Seq Data Mapping to the Genome

We downloaded tomato RNA-seq sequence data from the NCBI SRA database^2^ using SRA Toolkit. The RNA-seq data were retrieved from 27 published papers, which were listed in Supplementary Table S1. In total, 2,543 Gbs RNA-seq data were downloaded. The data from each publication were processed individually. The RNA-seq reads were mapped to tomato genome sequences using TopHat (v2.2.6) with default parameters (Kim et al., 2013). Then the transcript alignment file together with the ITAG3.20 annotation was used as input for Cufflinks (v2.2.1)^3^ (Trapnell et al., 2010). The GTF (Gene Transfer Format) files generated from each RNA-seq dataset after Cufflinks were merged using Cuffcompare script within the Cufflinks package (Trapnell et al., 2010). The GTF file generated from merged RNA-seq GTF files then was further merged using Cuffcompare script with the GTF file that was generated by the ASFinder for mapping the assembled ESTs and mRNA (transcripts) sequences to the genome to generate a final GTF file for AS analysis. AStalavista was used for AS event classification (Foissac and Sammeth, 2007).

### Transcript Functional Annotation

The sequences of the transcripts were retrieved using gtf_to_fasta tool in the tophat package (Kim et al., 2013), based on the GTF file generated by Cuffcompare program after merging the EST and mRNA mapping GTF file and RNA-seq mapping GTF file. They were functionally annotated using a procedure we reported previously (Min, 2018). The annotation information contains protein coding regions (ORF) predciton, assessment of full–length transcript coverage, protein family, and comparison with sequences of predicted gene models. Gene Ontology (GO) information was extracted also using a procedure reported previously (Min, 2018). Transcripts not having BLASTX hit against UniProtKB-SwissProt database were further used for non-coding RNA (ncRNA) identification by using BLASTN search against the non-coding RNA central database (version 10)^4^ with a cutoff *E*-value of 1e-5.

### Transposable Element Analysis in Introns

Intron sequences were retrieved using an in-house script. Transposable elements in the introns were identified using BLASTN searching against the RepBase (Bao et al., 2015)^5^ using a cutoff *E*-value of 1e-5.

### Internal Exon and Intron Length Distribution and Exon/Intron Junctions

The lengths of internal exons and introns in all transcripts and sizes of DNA fragments involved in AS were analyzed. The exon and intron junction sequences were extracted from genes not undergoing AS (non-AS genes) and genes undergoing AS (AS-genes). The exon/intron boundary sequence logo was created using the weblogo server^6^ (Crooks et al., 2004).

### Availability of Data

The assembled transcripts and AS events identified in this study along with the predicted gene models, along with the data reported previously in our group including *Brachypodium distachyon* (Sablok et al., 2011; Walters et al., 2013), pineapple (Wai et al., 2016), and sacred lotus (*Nelumbo nucifera*) (VanBuren et al., 2013), are available from plant alternative splicing database^7^ (Walters et al., 2013; VanBuren et al., 2013; Min et al., 2015; Wai et al., 2016, 2018; Sablok et al., 2017; Min, 2017, 2018). BLAST search is also available for searching the transcripts. The datasets for database construction and the supplementary data are publicly available at: http://proteomics.ysu.edu/publication/data/Tomato/.

## Results

### Features of Assembled Transcripts

In this work we integrated genome mapping data from available ESTs and mRNAs in the public nucleotide database with RNA-seq data downloaded from the NCBI SRA database that were obtained from 27 previous publications (see Supplementary Table S1). A total of 533,707 putative unique transcripts with an average length of 1,350 bp were obtained based on the final GTF file which was generated by merging the mapping GTF file of assembled EST and mRNA sequences and the mapping GTF file of RNA-seq data to tomato genome sequences (Table 1). The basic features of the transcripts in the dataset were summarized (Table 1).

**Table 1:** Basic features of the assembled unique transcripts in tomato plants.

Total unique transcripts	533707
Average transcript length (bp)	1350
Total genomic loci with at least one transcript	260681
Transcripts matching with gene model cDNAs	260365
Unique gene model cDNAs matching with transcripts	34522
Transcripts having a BLASTX match against Swiss-Prot database	226881
Total predicted ORFs from assembled transcripts	518307
Average length of predicted ORFs (amino acids)	215
Predicted full-length ORFs	182325
Predicted ORFs having a PFAM match	176234

The mapped transcripts were clustered to a total of 260,681 genomic loci (Table 1), which were significantly higher than the number of protein coding genes (35,768 gene models, 35768 protein sequences and 35,768 cDNAs) annotated in ITAG3.20. Using ungapped BLASTN search with 100% identity and a minimum length of 80 bp in a high score aligned segment, a total of 260,365 transcripts accounting for 48.8% of total transcripts matched with cDNA sequences of gene models. A total of 34,522 (96.5%) unique loci, represented by cDNA sequences, have matched at least one transcript. Assembled transcripts were annotated functionally using BLASTX search against the UniProt-SwissProt database. Among them, a total of 226,881 (42.5%) has a BLASTX hit and 182,325 transcripts were predicted to contain a complete ORF region, i.e., a full-length ORF. Within the assembled transcripts, a total of 518,307 ORFs were predicted with an average length of 215 amino acids and 176,324 ORFs were mapped to a protein family (Pfam) using rpsBLAST (Table 1). Transcripts which were not able to match with a predicted cDNA transcript sequence were likely novel transcripts. Whether the novel transcripts identified in the projects represent the transcription noise, i.e., without any biological significance, or play certain biological roles remain to be examined in future study.

### AS Events Identification

Tomato genome has 12 chromosomes (Tomato Genome Consortium, 2012). Chromosome zero (Chr0) is a segment of chromosome that has not been assigned to a specific chromosome (Table 2). There were 369,911 AS events identified from 34,419 genomic loci involving 161,913 transcripts. Although there are variations in the total number of AS events among different chromosomes, the general AS event distribution patterns are consistent across chromosomes (Table 2). Among the four basic AS types, IntronR is the prevalent type of AS event (18.9%), followed by AltA (12.9%) and AltD (7.3%), and ExonS as the least type (6.0%). Various complex types can be formed in transcript isoforms by combination of basic events and 54.9% of AS events were complex types (2).

**Table 2:** Summary of alternative splicing events in each chromosome of tomato plants.

	AltA (%)		AltD (%)		ExonS (%)		IntronR (%)		Others (%)		Total
Chr0	188	11.8	108	6.8	55	3.4	451	28.3	794	49.7	1596
Chr1	6410	13.5	3592	7.6	2923	6.2	9269	19.5	25276	53.2	47470
Chr2	5396	10.1	3156	5.9	2379	4.5	8324	15.6	34184	64.0	53439
Chr3	5168	13.2	2856	7.3	2274	5.8	7432	19.0	21476	54.8	39206
Chr4	3988	14.0	2228	7.8	1851	6.5	5567	19.5	14926	52.3	28560
Chr5	3026	13.4	1818	8.0	1434	6.3	4488	19.8	11883	52.5	22649
Chr6	4077	14.1	2173	7.5	1758	6.1	5938	20.5	15071	51.9	29017
Chr7	3379	12.3	1855	6.7	1661	6.0	5106	18.6	15506	56.4	27507
Chr8	3355	13.3	1839	7.3	1537	6.1	4979	19.8	13426	53.4	25136
Chr9	3281	12.8	1995	7.8	1600	6.3	4896	19.2	13786	53.9	25558
Chr10	2883	13.6	1572	7.4	1378	6.5	4509	21.3	10811	51.1	21153
Chr11	3254	13.0	1876	7.5	1630	6.5	4383	17.5	13973	55.6	25116
Chr12	3316	14.1	1905	8.1	1681	7.2	4693	20.0	11909	50.7	23504
**Total**	**47721**	**12.9**	**26973**	**7.3**	**22161**	**6.0**	**70035**	**18.9**	**203021**	**54.9**	**369911**

Among 369,911 transcripts generated from pre-mRNAs having AS, 150,131 matched to cDNAs of 23,233 unique annotated gene models (See Supplementary Table S2). We noticed that there were some gene models undergoing AS having only one corresponding transcript shown in the table because other transcripts involved in AS did not have a sufficient overlap region with the gene model transcript (Supplementary Table S2). However, the complete information of isoform transcripts can be obtained from the database and the genome browser. Based on the mapping analysis, there were 34,522 unique loci (gene models) having EST/mRNA or RNA-seq mapped, i.e., these genes were supported with transcription data. Thus, using only expressed genes the AS rate in tomato was estimated to be 67.3%. However, when all gene models were used, the estimated AS rate was 65.0%.

Li et al. (2014) reported that transposons were enriched in the retained introns in cotton plants. While only 2.9% of all introns contained transposable elements (TEs), 43% of the retained introns were found to have TEs in the AS transcripts, suggesting TE-insertion may result in IntronR during pre-mRNA splicing in cotton (Li et al., 2014). We retrieved 68,241 retained introns from our datasets with a length >50 bp and found only 812 TEs (1.2%). While in the whole set (138,127) of introns of the predicted gene models, using the same cutoff value, 3,105 TEs (2.3%) were found. Thus, the TE enrichment phenomenon reported by Li et al. (2014) was not found in tomato.

To facilitate identifying project specific AS events which may aid in elucidating the biological significances of AS isoforms, we analyzed AS events in each individual projects (Table 3). Because we used pooled data from different projects with variable data sizes and treatment conditions, it is difficult to directly compare the results from these projects (Supplementary Table S1). However, the overall trend in AS type frequency distribution is consistent with the final combined data (Tables 2, 3), that is, IntronR is the most prevalent type of AS, followed by AltA and AltD, with ExonR as the least frequent type. The RNA-seq mapping information including expression levels, AS event analysis, and information for sequence identifier mapping to the final assembled transcripts are available for downloading at http://proteomics.ysu.edu/publication/data/Tomato/projects/.

**Table 3:** Summary of alternative splicing events identified in EST and mRNA assembly dataset and each RNA-seq dataset in tomato plants.

Data^a^	AltA	AltD	ExonS	IntronR	Others
ESTs and mRNAs	13738	7779	9355	17255	32448
RNA-seq projects					
Alkan (2014)	2664	1841	1367	3966	3057
Chen (2013)	1425	1068	711	2238	1528
Cruz-Mendivil (2015)	122	130	88	329	448
Dai (2017)	2075	1440	992	2574	1920
Du (2015)	2557	1873	1273	3851	2878
Ezura (2017)	254	251	88	3698	1658
Gupta (2013)	2619	1909	1375	4723	3437
Higashi (2016)	1773	1256	967	7274	2916
Koenig (2013)	1791	1376	1005	2774	1998
Lopez-Casado (2011)	565	521	353	995	889
Shi (2013)	1773	1431	972	4806	2810
Shukla (2017)	18581	9519	7032	20625	29217
Sun (2015)	5309	3255	1876	5925	4530
Sundaresan (2016)	1857	1286	920	2461	1802
Tan (2015)	1268	954	656	1863	1287
Tang (2013)	1353	1057	816	1813	1401
Wang (2013)	5324	3022	1945	5945	4683
Wang (2016)	3603	2264	1857	5079	4818
Worley (2016)	1921	1308	1078	3769	2402
Xue (2017)	6455	3602	3246	8886	9113
Yang (2015)	2093	1356	1257	3259	2041
Ye (2015)	1389	1127	762	3678	2112
Zhang (2016)	6109	3399	2727	7825	6880
Zhang (2017)	2493	1623	1385	3121	2396
Zheng (2017)	412	376	267	972	683
Zouari (2014)	2679	1820	1436	3878	3091
Zouine (2014)	2853	1935	1357	3278	2453

*^a^The last name of the first author with year of the publication is used the project ID. The details of the reference for each project can be found in the Supplementary Table S1.*

### Functional Annotation of Transcripts

All transcripts were functionally annotated including BALSTX search against the UniProtKB-SwissProt database and predicting the ORF regions and completeness of ORFs (see section “Transcript Functional Annotation”). The predicted proteins were further annotated for the protein family (Pfam) analysis. To provide an overview of the protein family distribution in tomato proteome and proteins encoded by genes generating AS isoforms, we used the protein sequences of the gene models for Pfam analysis. Among 35,768 protein sequences of gene models a total of 22,322 entries had PFam matches with a total of 3,319 unique Pfam. Among a total of 23,233 protein sequences generated from AS genes, a total 16,531 had Pfam matches with a total of 3,114 unique Pfam. The top Pfam in the whole tomato proteome and proteins encoded by genes undergoing AS were listed in Table 4. The numbers of proteins in each Pfam varied significantly from 1 member in some families to 631 members in Pkinase (pfam00069). In average 74.1% of Pfam members were alternatively spliced with varying proportions in different protein families (Table 4). In considering the varying Pfam size, number of exons per gene and gene expression levels as well as functional differences of these protein coding genes, such a difference in AS rates in the genes belonging to different protein families is expected. Comparing with our previous Pfam analysis of proteins generated from AS genes in cereal plants and fruit plants, these Pfams found in tomato AS genes were also well conserved in other plant species (Min et al., 2015; Sablok et al., 2017).

**Table 4:** Protein families in gene models and alternatively spliced genes in tomato plants^∗^.

Pfam ID	Total	AS genes	%	Pfam abbreviation	Pfam description
pfam00069	631	503	79.7	Pkinase	Protein kinase domain
pfam07714	467	388	83.1	Pkinase_Tyr	Protein tyrosine kinase
pfam00067	337	216	64.1	p450	Cytochrome P450
pfam13041	316	247	78.2	PPR_2	PPR repeat family
pfam13639	223	140	62.8	zf-RING_2	Ring finger domain
pfam00931	217	151	69.6	NB-ARC	NB-ARC domain
pfam00076	179	162	90.5	RRM_1	RNA recognition motif
pfam00249	178	111	62.4	Myb_DNA-binding	Myb-like DNA-binding domain
pfam00201	169	86	50.9	UDPGT	UDP-glucoronosyl and UDP-glucosyl transferase
pfam10536	167	101	60.5	PMD	Plant mobile domain
pfam03171	147	87	59.2	2OG-FeII_Oxy	2OG-Fe(II) oxygenase superfamily
pfam00847	137	62	45.3	AP2	AP2 domain
pfam02519	130	35	26.9	Auxin_inducible	Auxin responsive protein
pfam00141	123	81	65.9	peroxidase	Peroxidase
pfam05699	118	28	23.7	Dimer_Tnp_hAT	hAT family C-terminal dimerisation
pfam00319	109	34	31.2	SRF-TF	SRF-type transcription factor
pfam02458	99	51	51.5	Transferase	Transferase family
pfam14432	95	68	71.6	DYW_deaminase	DYW family of nucleic acid deaminases
pfam00481	91	79	86.8	PP2C	Protein phosphatase 2C
pfam00854	86	75	87.2	PTR2	POT family
pfam01095	85	42	49.4	Pectinesterase	Pectinesterase
pfam00561	84	70	83.3	Abhydrolase_1	alpha/beta hydrolase fold
pfam00657	83	65	78.3	Lipase_GDSL	GDSL-like Lipase/Acylhydrolase
pfam00010	82	70	85.4	HLH	Helix-loop-helix DNA-binding domain
pfam03106	82	54	65.9	WRKY	WRKY DNA -binding domain
pfam01554	77	66	85.7	MatE	MatE
pfam13839	75	63	84.0	PC-Esterase	GDSL/SGNH-like Acyl-Esterase family found
pfam00004	74	64	86.5	AAA	ATPase family associated with various cellular
pfam02362	74	49	66.2	B3	B3 DNA binding domain
pfam00071	73	58	79.5	Ras	Ras family
pfam00083	73	53	72.6	Sugar_tr	Sugar (and other) transporter
pfam05695	72	13	18.1	DUF825	Plant protein of unknown function (DUF825)
pfam00082	71	41	57.7	Peptidase_S8	Subtilase family
pfam13499	71	40	56.3	EF-hand_7	EF-hand domain pair
Others	17227	13078	75.9		
**Total**	**22322**	**16531**	**74.1**		

The isoform transcripts generated by alternative pre-mRNA splicing may or may not be functional. Thus the impact of AS on the functionalities of isoforms was assessed using Pfam annotation information of the predicted proteins. Within a total of 369,911 pairs generating AS events, 114,729 (31.0%) pairs did not have Pfam annotation, 164,594 (44.5%) pairs had same Pfam annotation; 64,107 (17.3%) pairs had one isoform with Pfam annotation and one isoform did not have Pfam annotation, suggesting either no protein sequences predicted or a loss of protein functionality; and 26,481 (7.2%) pairs had different Pfam annotation, suggesting a functional domain change in the protein sequences resulting from AS. Thus, the comparative Pfam analysis of the proteins encoded by the isoform transcripts generated by AS revealed that about 24.5% of them may cause functional loss or change in the protein isoforms. Our previous analysis showed that the resulting functional domain loss or change by AS were 19.6% in maize, 20.9% in cotton, and 24.9% in pineapple, respectively (Wai et al., 2016; Min, 2017, 2018). The translation frame changes in AS isoforms is the main reason for protein domain loss or change.

A total of 342,073 transcripts not having a BLASTX hit against the UniProt Swiss-Prot database were further used to search the ncRNA database using BLASTN. The ncRNA database was obtained from the RNAcentral (release 10) with a total of 11,963,117 ncRNA sequences. With a cutoff *E*-value of 1e-5, we identified a total of 136,643 transcripts sharing similarities with known ncRNAs. The list of ncRNAs can be downloaded at http://proteomics.ysu.edu/publication/data/Tomato/.

### Gene Ontology (GO) Analysis

**FIGURE 1:**
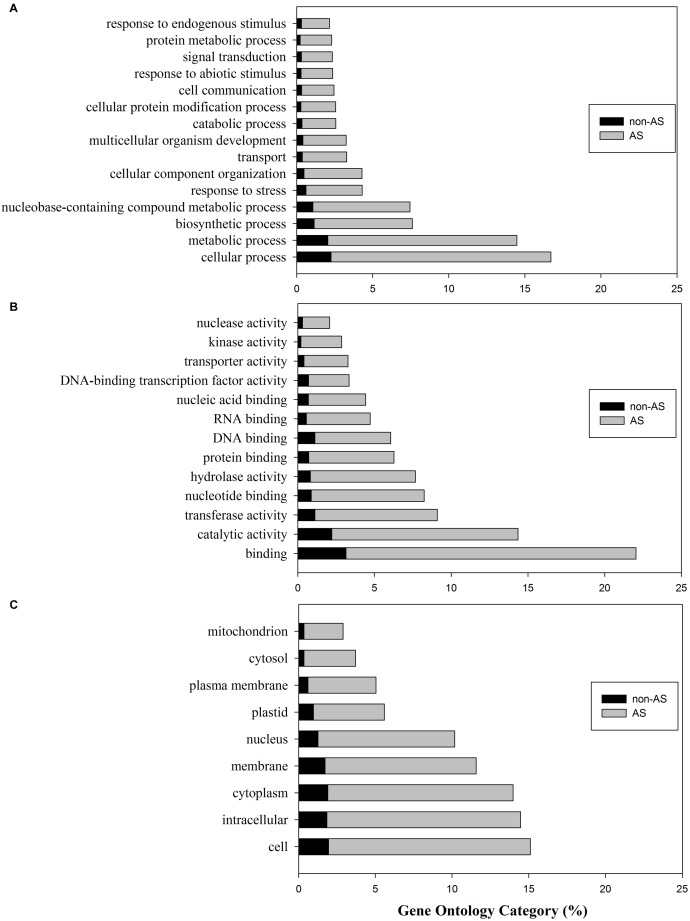
Gene ontology (GO) classification of tomato genes with pre-mRNAs not undergoing alternative splicing (non-AS genes) and genes with pre-mRNA undergoing alternative splicing (AS-genes). **(A)** Biological process; **(B)** Molecular function; **(C)** Cellular components.

The gene ontology^8^ is “a community-based bioinformatics resource that supplies information about gene product function using ontologies to represent biological knowledge” (Gene Ontology Consortium, 2014). GO classification has three major categories including the biological processes, molecular functions, and cellular components. We used the protein sequences of gene models to search the UniProtKB/Swiss-Prot database, then retrieved GO IDs based on the UniProt ID mapping table. Among 35,768 protein sequences predicted from gene models, 25,048 of them had a BLASTP hit with UniProtKB/Swiss-Protein dataset. Among them 18,031 were from protein sequences of genes with pre-mRNAs undergoing AS. We then retrieved 155,246 and 114,667 GO IDs for the whole set and AS gene set, respectively. The GO IDs were further mapped to each category using Slim Viewer with plant specific GO terms (McCarthy et al., 2006). The top GO terms in each category were presented in Figure 1. The percentage of each category was calculated based on the total counts in each category.

In the whole tomato proteome set, the mapped GO term counts were 47,795 in biological processes; 25,989 in molecular functions, and 33517 in cellular components. The comparative analysis showed that 86.5% of genes involved biological processes may undergo alternative splicing (Figure 1A). The top biological processes include cellular process, metabolic process, biosynthetic process, nucleobase-containing compound metabolic process, response to stress, cellular component organization, etc., (Figure 1A). In particular, 153 genes (76.5%) from a total of 200 genes involved in the secondary metablic process (GO:0019748) were found undergoing AS. These genes are involved in the biosynthesis of lycopene, carotenoid, abscisic acid, flavonoid, anthocyanins, etc., (Bovy et al., 2007; Liu et al., 2015). AS analyses involved in secondary metabolism pathways including the flavonoid pathway in tea plant have been carried out, suggesting AS may play important roles in regulation of flavonoids biosynthesis (Zhu J. et al., 2018; Qiao et al., 2019).

Similarly, the majority (86.2%) of tomato gene products in the category of molecular functions were also alternatively spliced (Figure 1B). The top categories of molecular functions include binding, catalytic activity, transferase, nucleotide binding, hydrolase activity, protein binding, etc., (Figure 1B). Cellular components analysis also revealed that ∼86.5% of tomato genes with GO cellular component annotation having pre-mRNAs were alternatively spliced (Figure 1C).

### Features of Exons, Introns and Exon-Intron Junctions

**FIGURE 2:**
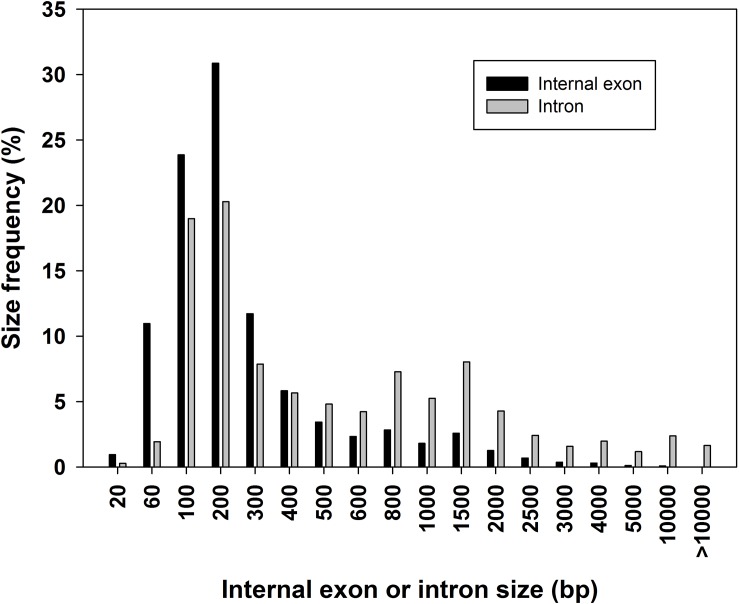
Distribution of internal exon size and intron size in tomato genes. Bin size are right inclusive (e.g., bin 100 comprises sequences of lengths 1–100 bp).

The lengths of internal exons and introns, as well as the DNA fragment sizes involved in AS events were calculated based on the RNA to genome mapping information (Table 5 and Figure 2). A total of 215,952 internal exons and 282,296 introns were extracted. The sizes of internal exons varied from 1 to 88,397 bp with a mean value of 282 bp; intron sizes varied from 5 to 313,176 bp with a mean value of 1,352 bp (Table 5). Among internal exons, 84.2% of them had a size of ≤400 bp and 94.6% were ≤1000 bp (Figure 2). In contrast, 55.0% of introns were ≤400 bp and 76.5% were ≤1000 bp. There were 0.01% of internal exons and 1.44% of introns ≥10 kb. In addition, there were 350 introns with a size >100 kb. As we removed alignments in the mapping of EST/mRNA assembled data, these introns were clearly from the RNA-seq mapping. We manually checked some of the transcripts having long introns and found that these extremely long introns were likely due to the mapping of the fused transcripts generated from different genes. The fused transcripts in plants often are ignored as transcription noise. However, it is known that fusion of transcripts in human is related to cancer development (Mertens et al., 2015; Kumar et al., 2016). Similar to what we found in *B. distachyon* and in fruit plants (Walters et al., 2013; Sablok et al., 2017), DNA fragments involved in AS events were relatively shorter than the average size of the internal exons or introns in tomato (Table 5). Thus, it is reasonable to conclude that small exons tend to be skipped and small introns tend to be retained.

**Table 5:** Summary of internal exon length and intron length of all transcripts, and DNA fragment sizes (bp) involved in alternative splicing events in tomato.

	Sample size	Length range (bp)	Mean (bp)	Standard deviation (bp)
Internal exons	215952	1–88397	282	547
Introns	282296	5–313176	1352	7609
Retained introns	70035	6–19337	366	710
Alternative acceptor sites	47721	1–12110	145	451
Alternative donor sites	26973	1–11238	212	493
Skipped exons	22161	2–25108	214	446

**FIGURE 3:**
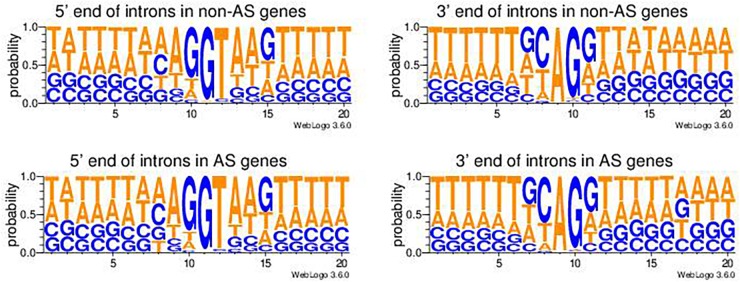
Pictograms of nucleotide probabilities at each position of the exon-intron junctions in genes not undergoing alternative splicing (non-AS genes) and genes undergoing alternative splicing (AS genes). The 5′-end intronic nucleotides are from position 11 to 20 in the left panel pictograms and the 3′-end intronic nucleotides are from position 1 to 10 in the right panel pictorgrams.

There were a total 46,114 introns extracted from genes not having AS (non-AS genes) and 236,182 introns from genes having AS (AS genes). Two nucleotides from each end of introns were extracted from both non-AS genes and AS genes. The majority of introns (90.6% in average) in both AS genes (90.9%) and non-AS genes (89.0%) had a canonical splicing junction site of 5′-GT..AG-3′ (Table 6). The minor types of splicing sites in introns included 5′-GC..AG-3′, 5′-GC..AT-3′, 5′-AT..AC-3′, and many others types (Table 6). A chi-square test showed there was no significant difference in the frequencies of the types of splicing sites between AS genes and non-AS genes (Table 6). The pictograms showed that the only noticeable difference in nucleotide usage probabilities in the junction sites at the 5′-end of introns between non-AS genes and AS-genes was position 8 (Figure 3, left panels). However, there were noticeable differences in the nucleotide probabilities at the 3′-end of introns but within the 5′-end of the next exonic region of, i.e., at position 14, 16, and 17 (Figure 3, right panels). Whether there is any biological significance remains to be examined.

**Table 6:** The usage of different splicing sites at both ends of the introns in tomato.

Types	Total	%	Non-AS gene	%	AS gene	%
5′-GT..AG-3′	255669	90.6	41053	89.0	214616	90.9
5′-GC..AG-3′	7571	2.7	1511	3.3	6060	2.6
5′-GC..AT-3′	3060	1.1	712	1.5	2348	1.0
5′-AT..AC-3′	2751	1.0	844	1.8	1907	0.8
5′-CT..AC-3′	2067	0.7	232	0.5	1835	0.8
5′-GT..AT-3′	2036	0.7	554	1.2	1482	0.6
Others	9142	3.2	1208	2.6	7934	3.4
Total	282296		46114		236182	

## Discussion

In this work a much higher number of transcripts, than any previous report, were identified in tomato, as we integrated all currently available EST/mRNA sequences with RNA-seq data generated from 27 RNA-seq projects covering a broad range of biological samples with plants grown under various conditions (see Supplementary Table S1). The human ENCODE project reported the genome is pervasively transcribed (∼76% of the full genome transcribed), and “many novel non-protein-coding transcripts have been identified, with many of these overlapping protein-coding loci and others located in regions of the genome previously thought to be transcriptionally silent” (Encode Project Consortium, 2007; Pennisi, 2012). Thus the current set of transcripts represents the most comprehensive and complete set of transcripts identified in tomato by now.

The basic type distribution patterns of AS events in tomato are consistent with findings in other plant species (Wang and Brendel, 2006; Walters et al., 2013; VanBuren et al., 2013; Thatcher et al., 2014; Min et al., 2015; Sablok et al., 2017). However, the proportion of the complex type is related to the transcriptome sampling size and thus the completeness and the average length of the transcripts (Min et al., 2015; Min, 2017, 2018; Sablok et al., 2017). Long transcripts have more exons covered and thus are able to detect AS isoforms having more than one type of AS event in their sequences. The estimated AS rate in tomato was estimated ∼65.0% in the analysis. This AS rate in tomato is nearly reached to the maximal rate, though such a value may never be able to obtain due to the dynamic nature of transcriptomes. At least this rate is comparable with the rate reported in Arabidopsis (∼60%) and in maize (55%) (Marquez et al., 2012; Mei et al., 2017; Min, 2017). Obtaining such a high rate is clearly due to relatively large number of RNA-seq data were used in current analysis. Using RNA-seq data to detect AS isoforms has been widely accepted as a suitable approach by the research community (Reddy et al., 2012; Sablok et al., 2013), and some of the identified isoforms have been experimentally validated using RT-PCR (Thatcher et al., 2014; Wang K. et al., 2016; Xu et al., 2016). As a meta-analysis in this work, we did not perform any validation on the identified isoforms generated by AS genes. In considering the dynamic nature of AS in responding to the changing environmental conditions and developmental regulations, however, experimental validations are needed in each specific experiment. Pacific BioSciences (PacBio) single-molecule real-time (SMRT) long-read isoform sequencing (Iso-Seq) and Nanopore sequencing from Oxford Nanopore Technologies (ONT) were two tools revolutionizing the way AS are identified (Zhao et al., 2019). The long reads sequencing technologies could avoid the error-prone step during transcripts assembly for RNA-seq reads from Illumina sequencing platform. In future, it will be interesting to validate these AS events based on RNA-seq short reads by using long reads from PacBio or ONT technologies. The list of potential isoforms identified in the work provides a foundation for designing experiments for exploring the biological significances of these AS events.

We also identified a large number of ncRNAs including miRNA and long ncRNAs. The ncRNAs play important regulatory roles in plant biology (Borges and Martienssen, 2015; Chekanova, 2015). A number of studies have reported the biological significances of ncRNAs in tomato plants (Wang et al., 2015, 2018b; Zhu et al., 2015). The list of ncRNAs compiled in the work will aid in further elucidating the roles of ncRNA playing in tomato biology.

## Data Availability

Publicly available datasets were analyzed in this study. This data can be found here: http://proteomics.ysu.edu/publication/data/Tomato/.

## Author Contributions

XM designed the experiments and performed the functional annotation. SC collected and processed the data for genome mapping. XM, FY, and LG analyzed the data. XM and LG wrote the manuscript.

## Conflict of Interest Statement

The authors declare that the research was conducted in the absence of any commercial or financial relationships that could be construed as a potential conflict of interest.

## Footnotes

1 https://solgenomics.net/organism/Solanum_lycopersicum/genome

2 https://www.ncbi.nlm.nih.gov/sra/docs/sradownload/

3 http://cole-trapnell-lab.github.io/cufflinks/

4 https://rnacentral.org/

5 https://www.girinst.org/repbase/

6 http://weblogo.threeplusone.com/

7 http://proteomics.ysu.edu/altsplice

8 http://www.geneontology.org
